# Decolorization and biodegradation of reactive sulfonated azo dyes by a newly isolated *Brevibacterium* sp. strain VN-15

**DOI:** 10.1186/2193-1801-1-37

**Published:** 2012-10-23

**Authors:** Elisangela Franciscon, Matthew James Grossman, Jonas Augusto Rizzato Paschoal, Felix Guillermo Reyes Reyes, Lucia Regina Durrant

**Affiliations:** Department of Food Science, Food Engineering School, University of Campinas, (UNICAMP) Rua Monteiro Lobato 80, Cidade Universitária Zeferino Vaz, Campinas, SP 13083-862 Brazil

**Keywords:** Azo dyes, Textile wastewater, Decolorization, Biodegradation, Detoxification, Brevibacterium, Tyrosinase, Carcinogenic aromatic amine

## Abstract

Azo dyes constitute the largest and most versatile class of synthetic dyes used in the textile, pharmaceutical, food and cosmetics industries and represent major components in wastewater from these industrial dying processes. Biological decolorization of azo dyes occurs efficiently under low oxygen to anaerobic conditions. However, this process results in the formation of toxic and carcinogenic amines that are resistant to further detoxification under low oxygen conditions. Moreover, the ability to detoxify these amines under aerobic conditions is not a wide spread metabolic activity. In this study we describe the use of *Brevibacterium* sp. strain VN-15, isolated from an activated sludge process of a textile company, for the sequential decolorization and detoxification of the azo dyes Reactive Yellow 107 (RY107), Reactive Black 5 (RB5), Reactive Red 198 (RR198) and Direct Blue 71 (DB71). Tyrosinase activity was observed during the biotreatment process suggesting the role of this enzyme in the decolorization and degradation process, but no-activity was observed for laccase and peroxidase. Toxicity, measured using *Daphnia magna,* was completely eliminated.

## Background

Azo dyes account for about one-half of all dyes produced and are the most commonly used synthetic dyes in the textile, food, paper making, color paper printing, leather and cosmetic industries (Chang and Lin 2001). The textile industry accounts for two-thirds of the total dyestuff market and during the dyeing process approximately 10% of the dyes used are released into the wastewater (Easton 1995). The amount of dye lost in industrial applications depends on the type of dye used and varies from 2% loss for basic dyes to about 50% loss for certain reactive sulfonated dyes when used with cellulosic fabrics due to the relatively low levels of dye fiber fixation (Shore 1995; McMullan et al. 2001; Pearce et al. 2003; Hai et al. 2007).

The high color content of dye process wastewater makes the presence of these dyes obvious and inhibits photosynthetic aquatic plants and algae by absorption of light, and as a result dye decolorization has been a primary goal of dye wastewater treatment processes (Banat et al. 1996). However, beyond color, the presence of these dyes in aqueous ecosystems presents serious environmental and health concerns as a result of the toxicity of the free dyes themselves and their transformation into toxic, mutagenic and carcinogenic amines, primarily as result of anaerobic microbial reductive cleavage of the azo bond (Chung and Cerniglia 1992; Weisburger 2002; Asad et al. 2007). In addition to toxicity, textile dye wastewaters have high TOC, high salt content and extremes in pH, with reactive dye baths having high pH and acid dye baths have low pH (Golob et al. 2005).

Dye wastewaters are treated physically and chemically by flocculation, coagulation, adsorption, membrane filtration, precipitation, irradiation, ozonization and Fenton’s oxidation (Lodha and Choudhari 2007; Wong et al. 2007). Although effective in dye removal, particularly for non-ionic dyes, these methods are expensive, add operational complexity to the process and can generate large amounts of dye contaminated sludge that must be disposed of, all of which add significantly to process costs (Lee 2000).

Due to the inherent drawbacks of physical, chemical and photochemical approaches to dye removal, the use of biological methods for the treatment of textile wastewaters has received attention as a more cost effective alternative (Olukanni Olukanni et al. 2006; Dos Santos et al. 2007). Anaerobic microbial wastewater treatment can be very effective in removing color, primarily by the activity of azo reductases that cleave the azo bond yielding the corresponding amines, which are frequently toxic, mutagenic and carcinogenic and resist further degradation under anaerobic conditions (Gottlieb et al. 2003; O’Neill et al. 2000; Pinheiro et al. 2004; Van der Zee et al. 2001; Van der Zee and Villaverde 2005). In this process the azo dye acts as the terminal electron acceptor in anaerobic respiratory oxidation of carbon sources and other electron donors (Carliell et al. 1995; Ryan et al. 2010).

In contrast, aerobic biological methods, such as activated sludge processes, are largely ineffective in the treatment of textile wastewaters as a stand alone process, resulting in little or no color removal from azo dyes, with dye removal primarily occurring through adsorption to the sludge (Nigam et al. 1996; Brik et al. 2006; Singh et al. 2007). Bacteria capable of aerobic decolorization and mineralization of dyes, specially sulfonated azo dyes, have proven difficult to isolate and the bacteria need to be specially adapted (McMullan et al. 2001; Pearce et al. 2003). However, both mixed and pure cultures of bacteria have been shown to be able to aerobically degrade and detoxify aromatic amines produced by anaerobic decolorization of azo dyes (Khalid et al. 2009).

The first azo reductases identified from anaerobic microorganisms were found to be oxygen sensitive, however, recent work described an *Enterococcus gallinarum* isolate, obtained from the effluent of a textile industry wastewater treatment plant, capable of decolorizing azo dye DB38 by azoreductase enzyme action under aerobic conditions (Amit et al. 2008). In another example, an azoreductase gene from *Bacillus latrosporus* RRK1, producing an azoreductase able to decolorize several azo dyes under aerobic conditions, was cloned and expressed in *E. coli,* which was then able to decolorize Remazol Red, and a level of 0.8 mg L^-1^ of dissolved oxygen was required (Sandhya et al. 2008).

A number of studies on the degradation of azo dyes by bacteria and fungi have indicated the involvement of extracellular oxidative enzymes such as, tyrosinase, lignin and manganese peroxidases and laccase (Fu and Viraraghavan 2001; Shanmugam et al. 2005; Zille et al. 2005; Ulson et al. Ulson de Souza et al. 2007; Kaushik and Malik 2009; Joshi et al. 2010; Kurade et al. 2011). Phenol oxidases, which can be divided into tyrosinases and laccases, are oxidoreductases that can catalyze the oxidation of phenolic and other aromatic compounds without the use of cofactors. In cultures which have shown the activity of these enzymes during dye degradation it has been observed that the dye structures can be cleaved symmetrically and asymmetrically (Duran et al. 2002; Dawkar et al. 2008; Dhanve et al. 2008).

Due to the recalcitrance of azo dyes to strictly aerobic conditions and the production and accumulation of environmentally deleterious amines under anaerobic conditions a combination of anaerobic decolorization followed by aerobic degradation of the amines to non-toxic products is considered most viable as a biological treatment scheme (Seshadri et al. 1994; Kudlich et al. 1996; Supaka et al. 2004). Moreover, the high salt content and extremes in pH associated with textile dye wastewaters have been shown to inhibit azo dye degradation by wastewater microbial communities indicating that tolerance to these conditions is also an important parameter to consider for efficient biotransformation of azo dye waste streams (Manu and Chauhari 2003; Sen and Demirer 2003; Asad et al. 2007). However, to achieve this, suitable strains of microorganisms must be identified and characterized to identify the best conditions for effective biological treatment of azo dyes.

Previous studies have shown that strains of *Brevibacterium* are able to degrade aromatic compounds including polynuclear aromatics, dibenzofurans and nitroaromatic compounds (Strubel et al. 1991; Stefan et al. 1994; Jain et al. 2005; Ningthoujam 2005; Onraedt et al. 2005). However, there is only one report of azo dye decolorization by *Brevibacterium* (Ng et al. 2010). In this case a *Brevibacterium casei*, isolated from sewage sludge from a dyeing factory, was shown to reduce Cr(VI) in the presence of azo dye Acid Orange 7 (AO7), possibly with the dye acting as the electron donor. Further degradation of the decolorized dye was not reported.

The present study was focused on the degradation of four reactive sulfonated azo dyes in a successive static/aerobic process using, exclusively, a *Brevibacterium sp.* isolated from a textile dye wastewater treatment plant. Dye decolorization was carried out under static conditions until no color was observed. The medium was then aerated by stirring to promote further degradation of the metabolites formed by cleavage of the azo bond into non-toxic metabolites. Tyrosinase, laccase and peroxidase enzyme activities as well as total organic carbon (TOC) were monitored during the biodegradation process. Biodegradation of the dyes was monitored for decolorization by UV–vis and degradation products were characterized using HPLC-MS. The effect of biotreatment on toxicity was evaluated using *Daphnia magna* the test organism.

## Results

### Strain isolation and characterization

Isolate VN-15 was initially isolated from activated sludge from a textile plant wastewater treatment facility based on its ability to produce large zones of decolorization around colonies grown on nutrient agar containing 100 L^-1^ of various azo dyes. The isolate is a Gram-positive rod, catalase and oxidase positive, nonfermentative, and oxidizes glucose to acidic products aerobically but not anaerobically. The 16S rRNA gene sequence of the VN-15 strain was determined and compared with 16S rRNA gene sequences in the Genbank nucleotide databases. The VN-15 strain was phylogenetically positioned in the genus *Brevibacterium*. The nucleotide alignment of the partial 16S rRNA gene sequence of this strain had identity values of 98 to 99%, with different Brevibacterium strains including the sequences of the strains *B. linens, B. permense, B. epidermidis* and *B. Iodinum*. The partial sequence determined in this study was deposited in the Genbank database under the accession number FJ598007 (B*revibacterium* sp. strain VN-15).

### Decolorization assays

**Figure 1:**
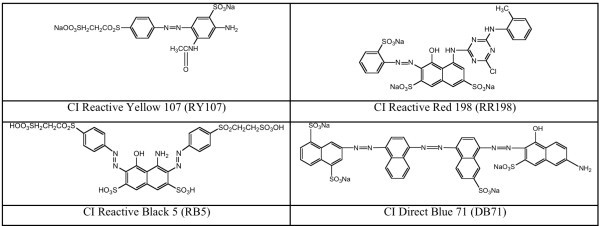
**Structure of azo dyes used in this study.**

The ability of *Brevibacterium sp.* (strain VN-15) to decolorize four azo dyes C.I. Reactive Yellow 107 (RY107), C.I. Reactive Red 198 (RR198), C.I. Reactive Black 5 (RB5), and C.I. Direct Blue 71 (DB71) was evaluated in a static/agitated sequential batch process as described in methods. The structures of the dyes are shown in Figure 1.

*Brevibacterium sp.* VN-15 could decolorize the dyes efficiently only when the medium was supplemented with carbon sources (data not shown). The medium that promoted the most rapid and efficient decolorization (>95%) under static conditions for all azo dyes was MM containing 3g L^-1^glucose and 1g L^-1^ sodium pyruvate (MMR) and this medium was used for all subsequent experiments for determining enzyme activities, degradation products and toxicity reduction. When sodium pyruvate was substituted by yeast extract (1g L^-1^) a similar rate of decolorization was observed. When glucose alone was added at a concentration of 1 or 3 g L^-1^ the rate of dye decolorization was reduced resulting in only 30 and 50% color removal after 168 h, respectively (data not shown). In MMR the monoazo dyes (RY107 and RR198) were decolorized to their maximum extent after 96 and 120 h respectively, while the more complex diazo RB5 and triazo DB71 dyes required 144 to 168 h, respectively, for maximum decolorization (Table 1). Subsequent aeration via stirring for a period of 168 h resulted in greater than 99% decolorization of all dyes being.(Table 1).

**Table 1:** **Azo dye decolorization by*Brevibacterium sp.*strain VN- 15 under static and aerobic conditions in the presence of 100 mg L**^**-1**^**of azo dyes and 3g L**^**-1**^**glucose and 1g L**^**-1**^**pyruvate**

	Decolorization time (h)	Decolorization (% of uninoculated control)	
Dyes	Static	Static	Aerobic
RY107	96 ± 1	98 ± 0.5	99 ± 0.3
RR198	120 ± 2	97 ± 0.2	99 ± 0.4
RB5	144 ± 2	95 ± 0.3	99 ± 0.2
DB71	168 ± 3	94 ± 0.1	99 ± 0.5

### Determination of enzyme activities

**Figure 2:**
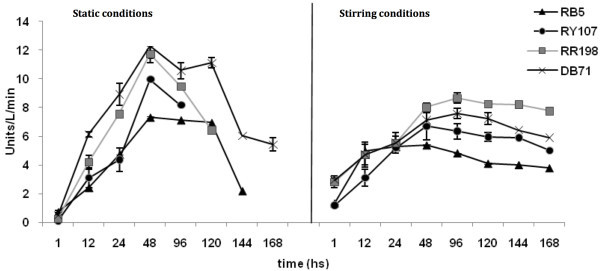
**Time course of tyrosinase activity in Brevibacterium sp. strain VN-15 cultures during biodegradation of azo dyes.**

The activities of the oxidoreductase enzymes (peroxidase, laccase and tyrosinase) were measured during the decolorization process in MMR. Static conditions were maintained until complete decolorization occurred as indicated in Table 1. This was followed by an aeration phase for and additional 168h. In the static and stirring conditions laccase and peroxidase activities were very low and likely did not play much of a role in dye decolorization/degradation. However, tyrosinase activity was observed both under static and aerated conditions (Figure 2). Under static conditions the activity increased until decolorization was complete and then decreased over of time for all dyes. Following initiation of aerated conditions the tyrosinase activity increased again for all dye cultures.

### UV–vis characterization

**Figure 3:**
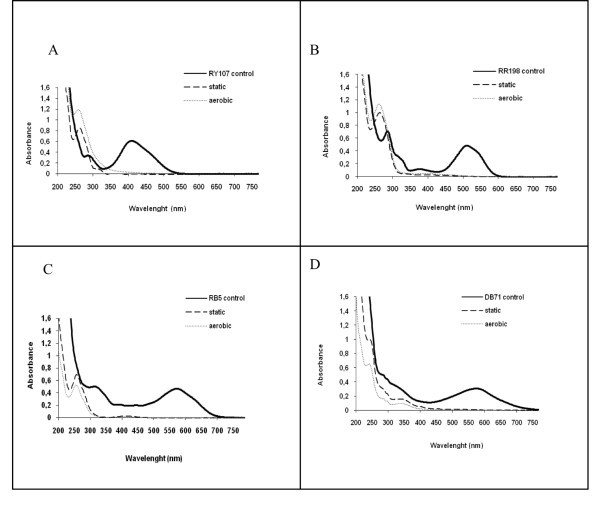
**UV–vis spectra of the azo dyes - Before (straight line) and after microaerophilic (dashed line) and aerobic (dotted line) treatments - A: RY107; B: RR198; C: RB5; D: DB71.**

As shown in Figure 3(a-d), virtually complete decolorization of all four azo dyes occurred under static conditions as shown by the disappearance of, the absorbance peaks in the visible region (390 to 750 nm). In the UV spectra of samples taken during static conditions, the absorbance observed in the 280–350 nm absorption profiles of all of the dyes diminished and were replaced by a new peak at 260 nm, which then either increased or diminished, depending on the dye, under aerated conditions.

### HPLC-MS analyses of RR198 dye biodegradation products

The biodegradation products of the RR198 dye produced under static and aerated conditions were analyzed by HPLC-MS for tentative identification of the unknown compounds. Out of forty possible compounds identified as metabolites from RR198 dye biodegradation only three had good matches to structures clearly consistent with the structure of RR198 (Table 2). These tentatively identified degradation products indicate that RR198 was cleaved at the azo bond as well as the amine bond between the naphthalene group and triazenic ring and at one of the sulfonate bonds producing the identified compounds 4-chloro-N-o-tolyl-1,3,5-triazin-2-amine; sodium 4-aminonaphthalene-2-sulfonate and 3,6-dimethyl-7-(o-tolyldiazenyl) naphthalen-1-amine.

**Table 2:** **Compound matches identified by the Cambridge SoftChemOffice program based on the RR198 dye structure**

Chemical name	Chemical formula	Molecular weight	Structure
4-chloro-N-o-tolyl-1,3,5-triazin-2-amine	C_10_H_9_ClN_4_	220	
sodium 4-aminonaphthalene-2-sulfonate	C_10_H_8_NNaO_3_S	245	
3,6-dimethyl-7-(o-tolyldiazenyl)naphthalen-1-amine	C_19_H_18_N_3_	289	

### Toxicity test and TOC reduction

The TOC of the medium was 2000 mg L^-1^ with the portion attributable to the azo dyes equal to 60 mg L^-1^. After decolorization (for the times indicated in Table 2) under microaerophilic (static) conditions the TOC was reduced from between 65% to 82% for all dyes, and after susequent aeration, for and additional 168 h, the TOC was further reduced (Table 3). The extent of the reduction in TOC demonstrates that the carbon sources were being consumed during the dye degradation process in both the static and aerobic phases.

**Table 3:** **Mortality of*Daphnia magna*exposed to a 1:4 dilution of culture supernatants containing azo dyes and the % TOC removal after incubation with*Brevibacterium*sp. strain VN-15 under static and aerobic conditions**

Dyes	Mortality (%)^*^			TOC reduction (%)^**^	
	Control	Static	Aerobic	Static	Aerobic
RY107	40	9	0	75	87
RB5	40	13	0	70	83
RR198	47	10	0	82	85
DB71	47	13	0	65	76

^*^S.D. ± 11% for all the data.

^**^S.D. ± 2% for all the data.

The results for *Daphnia magna* toxicity tests are presented as the percentage of death in the presence of samples taken from the static and stirring treatments compared to controls composed of the dye culture medium without bacterial treatment (Table 3). The tests were carried out in a 1:4 dilution of the original supernatant concentration because 100% mortality occurred in the undiluted and 1:2 diluted dye media. The uninoculated controls showed mortality between 40 to 47% at a dilution of 1:4. Samples taken from static cultures had mortality values much lower for all the dyes (≤ 13%), and samples from stirred cultures had no detectable toxicity for any dyes.

## Discussion

Based on the biochemical tests performed in this study, strain VN-15 was identified as *B. iodinum* due to the differentiating characteristic in the oxidase test (Table 1). However, several reclassifications have been made and the genus has been restricted to those bacteria with a close resemblance to the type species *B. linens* based on 16S rRNA gene sequence analysis and DNA-DNA hybridization experiments; twelve species are currently classified in this genus (Euzeby 2004). *Brevibacterium* are Gram-positive chemoorganotrophic, obligately aerobic and halotolerant to halophilic (typically growing well in 8% NaCl with some able to grow in 15% NaCl), and found in a wide range of habitats, especially those having high salt concentration (Jones and Keddie 1986). Textile effluents have elevated amount of salts, which may explain the presence of this bacterium.

*Brevibacterium* sp. strain VN-15 completely decolorized four azo dyes (Reactive Yellow 107, Reactive Red 198, Reactive Black 5 and Direct Blue 71) in a static/agitated sequential process only in the presence of a carbon source (glucose, pyruvate or yeast extract). In these experiments MMR, containing 3 gL^-1^ glucose and 1 gL^-1^ pyruvate as carbon sources, produced the best results. However, yeast extract, which has been the most commonly used a nutrient additive for dye bio-decolorization processes (Robert et al. 1998; Mohana et al. 2008), could be substituted for pyruvate with similar results. Previous studies have shown that pyruvate is able to enhance the degradation of aromatic compounds (Chung and King 2001). Azo bond reduction of the azo dye amaranth by *Shewanella decolorationis* S12 was most effective using lactate or formate as electron donors, while pyruvate also increased the reduction of amaranth but to a lesser extent (Hong et al. 2008).

The chemical structures of the dyes also influence the decolorization rates (Pasti-Grigsby et al. 1992). Dyes with simple structures and low molecular weights usually exhibit higher rates of color removal, whereas color removal is less efficient with highly substituted and higher molecular weight dyes (Pearce et al. 2003). Consistent with these observations, *Brevibacterium* sp. strain VN-15 required 96 h to decolorize the mono azo dye RY107 (the least substituted and least structurally complex dye used in this study) in comparison to 120 h for the highly substituted mono azo dye RR198, and 144 h for the diazo dye RB5 and 168 h for the triazo DB71.

Induction of tyrosinase activity for all dyes under static conditions for up to 96–120 h suggests this enzyme was involved in the decolorization process. In addition, under aerated conditions, the activity also increased and remained increased for the entire 168 h aeration period suggesting that tyrosinase could be involved in further biodegradation of the decolorized azo dye metabolites as well. In contrast, significant peroxidase or laccase activity was not detected. In recent studies, induction of the oxidative enzymes lignin peroxidase, laccase and tyrosinase was observed during the decolorization of sulfonated azo dyes by the bacteria *P. desmolyticum* and an *Exiguobacterium* sp. (Kalme et al. 2007a, 2007b; Dhanve et al. 2008). While in another report on dye decolorization by *Comamonas* sp. UVS, the induction of laccase and lignin peroxidase was observed but tyrosinase activity was not (Umesh et al. 2008).

Phenol oxidases, which can be divided into tyrosinases and laccases, are oxidoreductases that can catalyze the oxidation of phenolic and other aromatic compounds without the use of cofactors (Duran et al. 2002; Liu et al. 2004). Tyrosinases use molecular oxygen to catalyze two different enzymatic reactions: (I) the ortho-hydroxylation of monophenols to o-diphenols (monophenolase, cresolase activity) and (II) the oxidation of o-diphenols to o-quinones (diphenolase, catecholase activity) (Toussaint and Lerch 1987; Lerch 1995). However, aromatic amines and o-aminophenols have also been recognized as tyrosinase substrates that undergo similar ortho-hydroxylation and oxidation reactions (Claus and Filip 1990; Gasowska et al. 2004). In addition, oxidation products of tyrosinase can be further converted into heavier oligomeric species (Duran and Esposito 2000).

Under static conditions the UV–Vis spectroscopy demonstrated a decrease in absorbance between 280–350 nm (consistent with substituted benzene and naphthalene compounds) was observed for all dyes, with the corresponding formation of a new peak at 260 nm. The decrease in the absorbance of the peak between 280–350 nm and the formation of a new peaks at 260 nm suggest that there were changes to substituents on the aromatic groups, consistent with azo bond cleavage and other transformational changes to the aromatic structure. Aromatic amine spectra show extensive structure in the 260–300 nm range, and removal of absorption in the visible range and the production of absorption peaks around 260 nm has been observed with azo dyes chemically reduced with sodium sulfide and after their biological reduction with activated sludge under anaerobic conditions (Pinheiro et al. 2004). The peaks at 260 nm can be associated with the presence of oxidized aromatics such as phenolic and naphthoquinone compounds (Mielgo et al. 2001; Hsueh and Chen 2008). In addition, the absorbance peak at 260 nm can be attributed to the absorption by diketones (Lagesson et al. 2000). These compounds may be generated during bacterial degradation after cleavage of aromatic rings under aerobic conditions. After subsequent aeration the absorbance in the 260 region increased for the mono azo dye RY107 and for the highly substituted mono azo dye RR198, while the absorbance in this region decreased for the diazo dye RB5 and 168 h for the triazo DB71. The reason for this difference may be due to differences in the rates of transformation of the decolorized products during this phase. The increase in absorbance in the 260 nm region for RY107 and RR198 may then be attributable to a greater rate of additional transformation to intermediate products with absorbance in this region, resulting in their accumulation at greater rate than that for the other dyes.

The biodegradation products of the RR198 dye were analyzed by HPLC-MS for tentative identification of the unknown metabolites. Between possible compounds of the biodegradation RR198 azo dyes, three had reasonable matches in relation to metabolites presents in the sample: 4-chloro-N-o-tolyl-1,3,5-triazin-2-amine; sodium 4-aminonaphthalene-2-sulfonate and 3,6-dimethyl-7-(o-tolyldiazenyl) naphthalen-1-amine. After aerobic conditions the concentration of these metabolites was reduced indicating the were degraded further. The formation of these products would require the cleavage of azo bonds and sulfonate bonds. Cleavage of both the azo bond and the sulfonate bond was observed with the degradation of the diazo dye Reactive blue 172 by *Exiguobacterium* sp. RD3 (isolated from the dyestuff contaminated soil), which also expressed lignin peroxidase and laccase activity during the degradation process (Dhanve et al. 2008).

In addition to decolorization and degradation of the azo dyes, *Brevibacterium* sp. strain VN-15 also dramatically reduced the toxicity of the dye solutions after the static phase of incubation. Moreover, subsequent aeration of the static cultures further reduced toxicity below detectable levels. The present study demonstrates that *Brevibacterium* sp. strain VN −15 has the potential to decolorize and degrade toxic azo dyes to nontoxic products. The detection of tyrosinase activity throughout static and stirred stages indicates tyrosinase was involved in the biodegradation of the azo dyes. The addition of carbon substrates in the form of glucose, pyruvate or yeast exact was required for efficient decolorization indicating the dyes may be being used as terminal electron acceptors during their degradation.

This is the first report demonstrating effective decolorization and detoxification of azo dyes by a *Brevibacterium*.

## Methods

### Chemicals and culture medium

The azo dyes C.I. Reactive Yellow 107 (RY107), C.I. Reactive Red 198 (RR198), C.I. Reactive Black 5 (RB5), and C.I. Direct Blue 71 (DB71) were kindly provided by a plastic and textile company in Brazil. All other reagents were analytical grade and purchased from Sigma and used without further purification. Mineral salts medium (MM), pH 7, was prepared as previously described (Franciscon et al. 2009a, 2009b). To evaluate the effect of different carbon sources on dye decolorization MM was supplemented with the indicated amounts of glucose, sodium pyruvate and/or yeast extract, and 100 mg/L of dye. The highest degree and rate of decolorization occurred using MM supplemented with 3g/l glucose and 1g/l pyruvate, and this medium was used for all subsequent biodegradation experiments and was designated MM rich mineral medium (MMR).

### Strain isolation and characterization

*Brevibacterium sp.* strain VN-15 was isolated from activated sludge obtained from the Vicunha Textile Company, Itatiba, Brazil. Serial dilutions (10^-1^ to 10^-6^) of the sludge were inoculated by the spread plate technique onto Nutrient Agar plates containing azo dyes (100 mg L^-1^) and incubated under low oxygen conditions. *Brevibacterium sp.* strain VN-15 was chosen for further evaluation based the production of a large decolorization zone in the azo dye containing plates. The strain was maintained on slants of Nutrient Agar.

The identification of *Brevibacterium sp.* strain VN-15 was based on standard morphological and biochemical methods as described by Benson (1985), and 16S rRNA gene sequence analysis. Genomic DNA was obtained according to Ausubel et al. ( 1989). The 16S rRNA gene was amplified by PCR using the bacteria specific primers, 27f and 1401r (Lane 1991).

### Dye decolorization

Decolorization experiments for each dye were performed by growing *Brevibacterium sp.* strain VN-15 in 500 mL Erlenmeyer flasks containing 350 mL of sterile MM supplemented with 100 mg L^-1^ of dyes plus the indicated carbon source. Cultures were first incubated under static conditions to provide conditions of oxygen limitation, at 30°C, for the indicated times, until decolorization was complete. All cultures was then aerated by stirring for 168 h without any further supplementations to the medium. Dye decolorization was evaluated by measuring the change in absorbance between 200 and 800 nm with a Shimadzu 2101 UV-visible spectrophotometer in the static and aerobic stages.

### Preparation of enzyme extract

*Brevibacterium sp.* strain VN-15 was grown in in 500 mL Erlenmeyer flasks containing 350 mL of sterile MMR, containing 100 mg L^-1^ dyes and inoculated with a 3% by volume culture of *Brevibacterium sp.* strain VN-15 previously grown for 24 h in MMR without dyes. Samples were harvested and the cells were sonicated with 5 seconds pulses separated by 2 minutes intervals repeated 8 times (Sharp Silent Sonic Model UT-204). The sonicator was maintained at an amplitude output of 40 and a temperature of 4°C (Parshetti et al. 2006). The sonicated cells were then centrifuged at 20,000 x g for 15 min, to remove cell debris, and the supernatant was used as the source of enzyme.

### Determination of enzyme activity

Tyrosinase enzyme activity was assayed as described by Kamahldin and Eng (2003). Tyrosinase activity was determined using 0.1 mL of 1mM tyrosine solution (1 M phosphate buffer at pH 7) as substrate, 0.6 mL of the enzyme preparation and 0.3 mL of distilled water in a final volume of 1 mL. The oxidation of tyrosine to dihydroxyphenylalanine was monitored spectrophotometrically by measuring the increase in absorbance at 280 nm. One unit of tyrosinase activity was equal to a Δ280nm of 0.001 per min at pH 7.0 at 25°C in a 1.0 mL reaction mix containing 1mM tyrosine solution. Laccase activity was assessed using 0.1 mL of 0.5 mM syringaldazine solution in ethanol (due to its limited solubility in aqueous solutions) as substrate, 0.2 mL of 0.05 M citrate phosphate at pH 5, 0.6 mL of enzyme preparation and 0.1 mL of distilled water in a final volume of 1 mL (Szklarz et al. 1989). The oxidation of syringaldazine was monitored spectrophotometrically at 525 nm. Peroxidase activity was monitored using the same substrate used for laccase with 0.1 mL of 2mM hydrogen peroxide solution instead of distilled water. All enzyme assays were run in triplicate.

### High performance liquid chromatography mass spectrometry analysis (HPLC-MS)

The biodegradation products of azo dye RR198 produced by *Brevibacterium sp.* strain VN-15 in MMR were analyzed by HPLC-MS. Culture samples were centrifuged (20,000 × g for 15 min) and filtered through a 0.25 μm pore size filter. Aliquots of 25 μL were injected into a HPLC-MS system consisting of an HPLC system (Waters, USA) coupled to a mass spectrometer with hybrid quadrupole (Q) and time-of-flight (ToF) mass analyzers from Micromass (Waters, USA), with an electrospray source interface (LC-ESI-MS-MS). Instrument control and data processing were carried out by Masslynx 4.0 software. The mobile phase components used were degassed in an ultrasonic bath (Model USC 700, Unique Thorton, Brazil) before use in the LC system.

A Varian reverse phase C_18_ HPLC column (150 × 2.1 mm, 5 μm particle size) was used to separate the biodegradation products. The column temperature was set at 25°C. The mobile phase was composed of water and methanol, using gradient elution. The gradient elution profile, using a flow rate of 0.2 ml min^-1^, consisted of (in percent by volume; duration (min)) water (100; 30), water:methanol (50:50; 3), ending with methanol (100; 2). The quadrupole analyzer was programmed to select ions with m/z in the range from 50 to 1200 u. The ionization conditions selected were: cone gas flow (150 L h^-1^), desolvation gas flow (350 L h^-1^), polarity (ESI+), capillary energy (2900 V), sample cone energy (30 V), extraction cone energy (2.0 V), desolvation temperature (350°C), source temperature (120°C), ionization energy (2.0 V), collision energy (4 V), and multi-channel plate detector energy (2700 V).

Tentative identification of the metabolites from azo dye biodegradation was obtained by comparing the acquired mass spectra to spectra in the MS Database using the Cambridge SoftChem Office 2008 program.

### TOC measurement

The change in organic carbon of the biotreated azo dye cultures was monitored by measuring the Total Organic Carbon (TOC) using a TOC analyzer (Shimadzu 5000A) as previously described in (Franciscon et al. 2009a, 2009b).

### Toxicity test

*Daphnia magna* is a commonly used bioindicator test aquatic organism in acute and chronic toxicity studies of chemical compounds present in aquatic ecosystems (USEPA 1985). The acute toxicity tests using *D. magna* were carried as previously described (Franciscon et al. 2009a, 2009b).
